# Supplementary material for: Quantitative Examination of Five Stochastic Cell-Cycle and Cell-Size Control Models for Escherichia coli and Bacillus subtilis
Source: Front Microbiol. 2021 Oct 26;12:721899. doi: 10.3389/fmicb.2021.721899 (PMC8594374; doi:10.3389/fmicb.2021.721899)
Supplement: Supplementary file 1 [file Data_Sheet_1.pdf]

SUPPLEMENTARY MATERIAL

Quantitative examination of five stochastic cell-cycle and cell-size control models for *Escherichia coli* and *Bacillus subtilis*

Guillaume Le Treut, Fangwei Si, Dongyang Li, and Suckjoon Jun

CONTENT

Supplementary Methods **2**

Strains and growth conditions 2

Microfluidics, Microscopy and Image processing 2

Data availability 2

I-value analysis 2

Simulations 3

Supplementary Figures **5**

Supplementary Appendices **6**

Appendix A: Cell size homeostasis in the RDA model 6

Appendix B: Calculation of ρ(si, δid) for the IDA model 6

Appendix C: Calculation of ρ(si, Sd) and ρ(Sd(n), Sd(n+1)) for the sHC and IA models 7

Appendix D: Calculation of ρ(si, Sd) and ρ(Sd(n), Sd(n+1)) for the CCCP model 8

Supplementary Methods

Strains and growth conditions

The following strains were used in this study.

- BW25113: F- DE(araD-araB)567 lacZ4787(del)::rrnB-3 LAM- rph-1 DE(rhaD-rhaB)568 hsdR514.

(Note that the genotype of BW27378 is F- DE(araD-araB)567 lacZ4787(del)::rrnB-3 LAM- rph-1 DE(rhaD-rhaB)568 hsdR514 DE(araH-araF)570(::FRT).)

- SJ_FS130: To construct this strain, we introduced ΔdnaN::[dnaN-ypet] into BW25113 using P1 transduction.
- SJ_DL188: MG1655 F- λ- rph-1 dnaA msfGFP kan mCherry-dnaN

We used minimal MOPS media for the MG1655 experiments and minimal M9 glucose media with and without uracil for the SJ_FS130 (BW25113 based) experiments.

Microfluidics, Microscopy and Image processing

We used the same method as described in (11).

Data availability

The scripts used to produce the results presented in this manuscript are available in the branch `frontiers` of the repository: https://github.com/junlabucsd/DoubleAdderArticle/tree/frontiers. The repository also contains a copy of all the experimental data used in this manuscript. Please start at the repository index (https://github.com/junlabucsd/DoubleAdderArticle/blob/frontiers/Response/frontiers_index.ipynb). The commit at the date of publication of the manuscript is `4d3b79dc8492324f2ee4fc789d6e9a24a8c98c82`.

I-value analysis

Following the methodology proposed by Witz *et al.* (17), we first defined the covariance matrix (Eq. 13) of the analyzed variables. Then the *I*-value was computed as:

$$I = \frac{det(K)}{\prod_i k_{ii}},$$

where the k_{ii} are the diagonal elements of the covariance matrix, hence the variances. To compute the *I*-values of the four models listed in Figure 3 (top), we used the following combinations of physiological variables:

**Model**	**Variables**
RDA	$\lambda, \delta_{id}, \delta_{ii}, s_i$
IDA	$\lambda, \Delta_d, \delta_{ii}, S_b$
IA	$\lambda, \delta_{ii}, \tau_{cyc}, s_i$
sHC	$\lambda, \tau_{cyc}, s_i, S_b$

In Figure 3(bottom), we defined 18 physiological variables in line with the definitions given by (17)(see Figure S1): λ, S_b, S_d, S_i, Δ_d, Δ_{bi}, s_i, $s_i^{(n-1)}$, $s_i^{(n+1)}$, δ_{ii}, δ_{id}, R_{ii}, R_{id}, R_{bd}, R_{bi}, τ, τ_{cyc} and τ_{ii}. We generated all possible combinations of 4 variables, that is = 3060. We emphasize the difference between s_i and S_i. The former is the cell size per origin of replication at the initiation event associated with the division in the current generation; therefore it could be in a previous generation (*e.g.* mother, grand-mother cell). The latter is the cell size when replication initiation occurs in the current generation. These 2 variables are only the same in the case of non-overlapping cell cycles (Figure 1). We also found that the results of this

analysis were sensitive to processing of the experimental data. In their work, Witz and colleagues, instead of using the measured values for S_b, S_d, S_i and s_i, first fitted traces of cell sizes to an exponential function and took values interpolated by this fit. Yet it did not affect the relative ranking of the division-centric model with respect to the initiation-centric model.

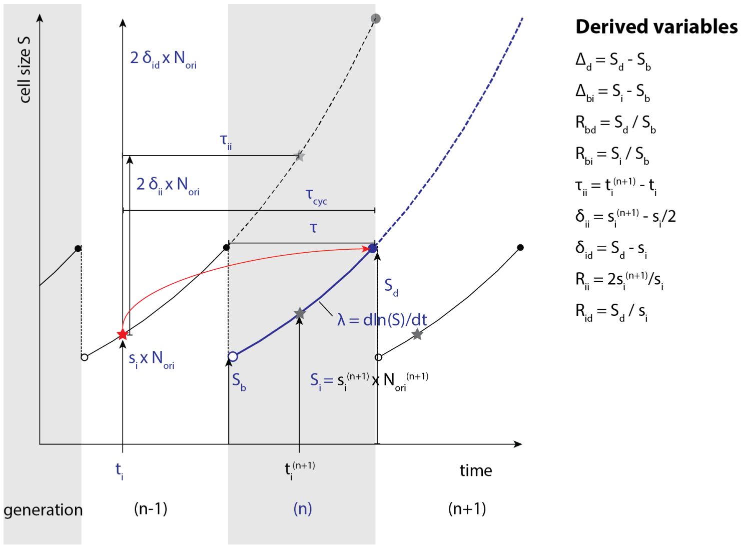

Figure S1: Definition of the physiological variables in a scenario with 2 overlapping cell cycles. All variables with blue font are associated with the current generation (*n*). We added a superscript when using physiological variables associated with another generation. We also defined variables derived from these quantities. Replication initiations are indicated with stars, and the red star is the initiation determining the division for the current generation. N_{ori} is the number of origins of replication just before initiation happens.

Simulations

In this study we have performed simulations of both initiation-centric and division-centric models. For this, we have re-used the code provided in (17). Few and minor modifications have been made, but these modifications did not affect the outcome or the essence of the original simulations. Simulations performed consist of:

- Repeats of the simulations performed by Witz and colleagues in their original study.
- Simulations using Witz and colleagues' original parameters, but with perfectly symmetrical division$_i$.
- Simulations of Witz et al. model, and our model, using experimental parameters taken from experimental datasets from Witz and et al. and datasets published in (11).

Witz et al.'s simulations of the RDA reproduced the adder behavior observed in their data, in apparent contradiction with our prediction in Eq. 5 that the RDA model is inconsistent with size homeostasis by the adder. We analyzed their simulations and found that they produced the adder-like behavior because of the additional fluctuations in the septum position (Figure S2a). Experimentally, septum position represents the most precise control among all measured single-cell parameters with CV < 5% (8, 28). Indeed, removing fluctuations in the septum position alone made the simulation deviate from experiment in a quantitative manner consistent with Eq. 5. We also conducted a similar analysis using our experimental data (11), and reached the same conclusion (Figure S2b). Based on this observation, we conclude that the RDA model does not self-consistently explain the adder phenotype.

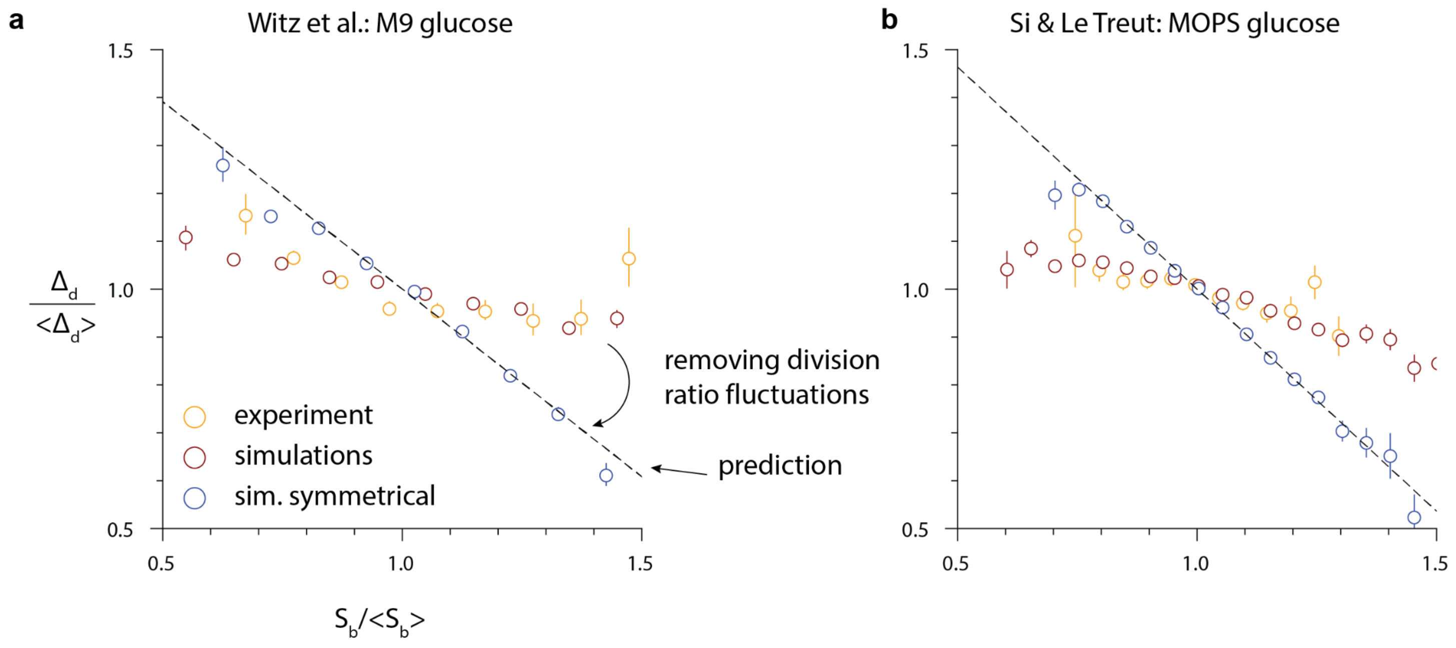

Figure S2: Agreement of the RDA model with experimental data from **a.** (17), M9 + glucose condition and **b.** (11), MOPS + glucose condition (MG1655 *E. coli*). After removing fluctuations in the division ratio, simulations don't agree with experimental data.

Supplementary Figures

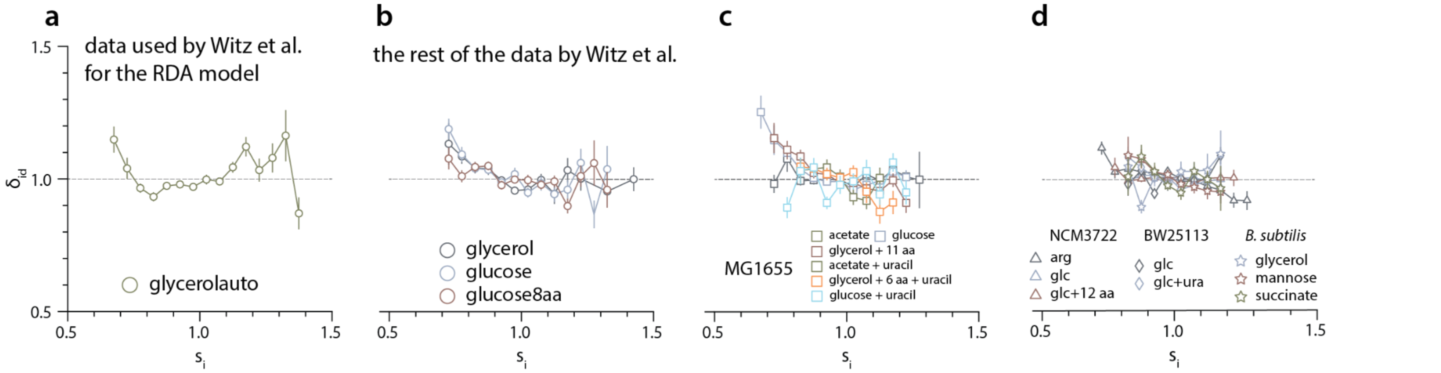

Figure S3 - a. Witz et al.'s validation of the RDA model is exclusively based on this data set. **b**. 3 out of the 4 datasets of Witz et al. present a slight negative correlation. **c-d**.Most of the experimental data we obtained with *E. coli* MG1655, NCM3722, BW25113 and *B. subtili*s NCIB 3610 strain also show varying degrees of negative correlation, consistent with the IDA model.

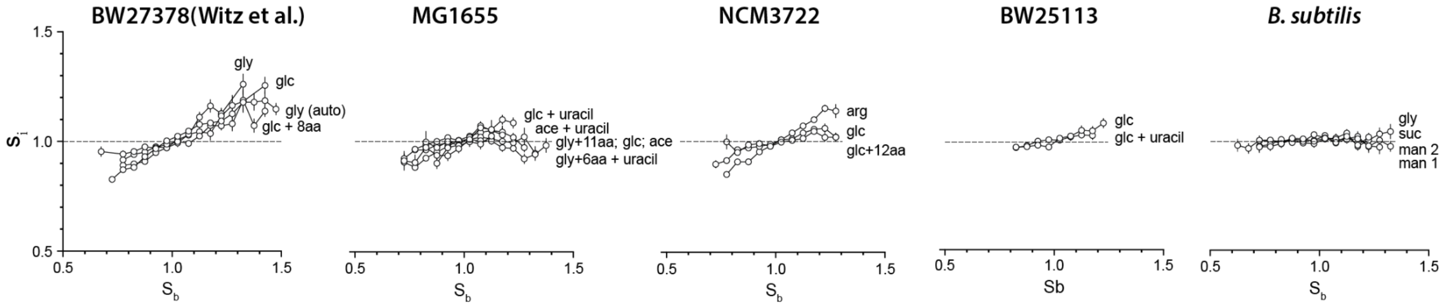

Figure S4 - Varying degrees of positive correlation between the birth size S_b and the initiation size per origin s_i. The experimental data from (17) shows the most positive correlation whereas experimental data acquired in our lab with *E. coli* MG1655 and with *B. subtilis* show moderate to zero correlation.

Supplementary Appendices

Appendix A: Cell size homeostasis in the RDA model

In the model proposed by Witz and colleagues, the cell size per origin at division is determined by the cell size at initiation per origin s_i, the added size per origin between consecutive replication initiation events δ_{ii}, and the added size per origin from replication initiation to cell division δ_{id}. The following relation holds:

$$S_d^{(n)} = s_i^{(n)} + 2\,\delta_{id}^{(n)},$$
$$s_i^{(n+1)} = \tfrac{1}{2} s_i^{(n)} + \delta_{ii}^{(n)},$$

where the index as n denotes the generation (or division cycle) . Under the assumption that $\delta_{ii}^{(n)}$ (resp. $\delta_{id}^{(n)}$) are independently and identically distributed Gaussian stochastic variables with mean μ_{ii} (resp. μ_{id}) and standard deviation σ_{ii} (resp. σ_{id}), it follows that $s_i^{(n)}$ and $S_d^{(n)}$ are also Gaussian stochastic variables. At large n, they converge to the limiting distributions $s_i \equiv N(\mu_i, \sigma_i)$ and $S_d \equiv N(\mu_d, \sigma_d)$, where:

$$\mu_i = 2\,\mu_{ii},\ \sigma_i^2 = \tfrac{4}{3}\sigma_{ii}^2.$$

$$\mu_d = 2\,(\mu_{ii} + \mu_{id}),\ \sigma_d^2 = 4\,\left(\tfrac{1}{3}\sigma_{ii}^2 + \sigma_{id}^2\right).$$

The mother-daughter correlation for division size is a central quantity in cell size homeostasis, which can be derived in this model. As a first step, let us define the centered variables: $ds_i^{(n)} = s_i^{(n)} - \mu_i$ and $dS_d^{(n)} = S_d^{(n)} - \mu_d$. We then obtain the relations:

$$< ds_i^{(n+1)} \cdot ds_i^{(n)} > \;=\; \tfrac{1}{2}\sigma_i^2,$$

$$< dS_d^{(n+1)} \cdot dS_d^{(n)} > \;=\; < ds_i^{(n+1)} \cdot ds_i^{(n)} >,$$

where the brackets denote averages. We therefore obtain the mother-daughter Pearson correlation coefficients (in the large n limit):

$$\varrho_i = \frac{<ds_i^{(n+1)} \cdot ds_i^{(n)}>}{\sigma_i^2} = \frac{1}{2}.$$

$$\varrho_d = \frac{<dS_d^{(n+1)} \cdot dS_d^{(n)}>}{\sigma_d^2} = \frac{1}{2}\left(1 + 3\frac{\sigma_{id}^2}{\sigma_{ii}^2}\right)^{-1}.$$

It also follows that the Pearson correlation coefficient between initiation size per origin and division size is:

$$\varrho_{id} = \frac{<ds_i^{(n)} \cdot dS_d^{(n)}>}{\sigma_d\,\sigma_i} = \left(1 + 3\frac{\sigma_{id}^2}{\sigma_{ii}^2}\right)^{-1/2}.$$

In this model the joint distribution ($S_d^{(n)}$, $S_d^{(n-1)}$) is a bivariate Gaussian, therefore we can write the conditional expectation of $S_d^{(n)}$ as:

$$< S_d^{(n)} | \, S_d^{(n-1)} > \;=\; \varrho_d\, S_d^{(n-1)} \;+\; (1 - \varrho_d)\,\mu_d.$$

With the hypothesis of symmetrical division, namely $S_b^{(n)} = 2\ S_d^{(n-1)}$, we obtain for the conditional expectation of the added size from birth to division:

$$< S_d - S_b \,|\, S_b > \;=\; (2\,\varrho_d - 1)\ S_b + (1 - \varrho_d)\ \mu_d.$$

Therefore, the “adder” principle is equivalent to having $\rho_d = 1/2$. Therefore, the RDA model always results in $2\,\rho_d - 1 < 0$. In fact, it only reproduces the “adder” principle in the deterministic limit $\sigma_{id} \rightarrow 0$.

Appendix B: Calculation of $\rho(s_i, \delta_{id})$ for the IDA model

In the IDA model, the cell size at division is determined by the cell size at birth S_b and the added size from birth to division Δ_d; and the cell size per origin at initiation in the next cell cycle $s_i^{(n+1)}$ is determined by the added size per origin between consecutive replication initiation events δ_{ii}, and the cell size per origin at initiation s_i. The following relations hold:

$$S_d^{(n)} = \tfrac{1}{2}S_d^{(n-1)} + \Delta_d^{(n)},$$
$$s_i^{(n+1)} = \tfrac{1}{2}s^{(n)} + \delta_{ii}^{(n)},$$

where the index n denotes the generation (or division cycle), and $\{\Delta_d^{(n)}\}$ and $\{\delta_{ii}^{(n)}\}$ are independently distributed random variables. Denoting $\mu_{dd} = <\Delta_d>$ and $\mu_{ii} = <\delta_{ii}>$, we have $\mu_d = <S_d> = 2\mu_{dd}$ and $\mu_i = <s_i> = 2\mu_{ii}$. We also define the centered variables: $d\Delta_d^{(n)} = \Delta_d^{(n)} - \mu_{dd}$, $dS_d^{(n)} = S_d^{(n)} - \mu_d$, $d\delta_{ii}^{(n)} = \delta_{ii}^{(n)} - \mu_{ii}$ and $ds_i^{(n)} = s_i^{(n)} - \mu_i$. Denoting $\sigma_{dd}^2 = <d\Delta_d^2>$ and $\sigma_{ii}^2 = <d\delta_{ii}^2>$, we have $\sigma_d^2 = <dS_d^2> = \tfrac{4}{3}\sigma_{dd}^2$ and $\sigma_i^2 = <ds_i^2> = \tfrac{4}{3}\sigma_{ii}^2$.

The added size between initiation and division reads $\delta_{id}^{(n)} = (S_d^{(n)} - s_i^{(n)})/2$, where the factor of 2 reflects the fact that origins of replication double at initiation. Therefore:

$$<ds_i^{(n)} \cdot d\delta_{id}^{(n)}> = -\tfrac{1}{2} <(ds_i^{(n)})^2>,$$

by independence of $S_d^{(n)}$ and $s_i^{(n)}$, and we obtain the Pearson correlation coefficient:

$$\frac{<ds_i^{(n)} \cdot d\delta_{id}^{(n)}>}{\sigma_i \cdot \sigma_{id}} = -\left(1 + \frac{\sigma_{dd}^2}{\sigma_{ii}^2}\right)^{-1/2}.$$

Appendix C: Calculation of $\rho(s_i, S_d)$ and $\rho(S_d^{(n)}, S_d^{(n+1)})$ for the sHC and IA models

Let us rewrite Eq. 1 as:

$$S_d^{(n)} = s_i^{(n)}\alpha^{(n)},$$

where $\alpha^{(n)} = \exp(\lambda^{(n)}\tau_{cyc}^{(n)})$. To derive $\rho(s_i, S_d)$ and $\rho(S_d^{(n)}, S_d^{(n+1)})$, we will assume that $\{s_i^{(n)}\}$ and $\{\alpha^{(n)}\}$ are independent random vectors. This is true in the IA model (because δ_{ii} is independent of the other physiological variables), but it is an additional constraint for the sHC model. Using this assumption, we can derive the mean and variance of the cell size at division. The mean is given by:

$$\mu_d = <S_d> = \mu_i\mu_\alpha,$$

where $\mu_i = <s_i>$ and $\mu_\alpha = <\alpha>$. Let us introduce the centered variables: $ds_i^{(n)} = s_i^{(n)} - \mu_i$, $d\alpha^{(n)} = \alpha^{(n)} - \mu_\alpha$ and $dS_d^{(n)} = S_d^{(n)} - \mu_d$, and the variances $\sigma_i^2 = <ds_i^2>$ and $\sigma_\alpha^2 = <d\alpha^2>$. The variance of the cell size at division is given by:

$$\sigma_d^2 = <dS_d^2> = <s_i^2><\alpha^2> - \mu_d^2 = \mu_d^2\left(\left(\frac{\sigma_i}{\mu_i}\right)^2 + \left(\frac{\sigma_\alpha}{\mu_\alpha}\right)^2 + \left(\frac{\sigma_i}{\mu_i}\right)^2\left(\frac{\sigma_\alpha}{\mu_\alpha}\right)^2\right).$$

We now compute $\rho(s_i, S_d)$:

$$\varrho_{id} = \frac{<ds_i \cdot dS_d>}{\sigma_i\sigma_d} = \frac{\sigma_i}{\sigma_d}\mu_\alpha = \frac{\eta_i}{\sqrt{\eta_i^2+\eta_\alpha^2+\eta_i^2\eta_\alpha^2}},$$

where we have introduced the coefficient of variations (CVs): $\eta_i = \sigma_i/\mu_i$ and $\eta_\alpha = \sigma_\alpha/\mu_\alpha$. As can be seen, the value of ρ_{id} depends on the CVs of the initiation size and of $\alpha = \exp(\lambda\tau_{cyc})$. When α is deterministic (*i.e.* η_α=0), division is "slaved" to replication initiation and ρ_{id}=1.

To compute $\rho(S_d^{(n)}, S_d^{(n+1)})$, we introduce the mother/daughter correlations:

$$\varrho_i = \frac{<ds_i^{(n)} \cdot ds_i^{(n+1)}>}{\sigma_i^2} \text{ and } \varrho_\alpha = \frac{<d\alpha^{(n)} \cdot d\alpha^{(n+1)}>}{\sigma_\alpha^2}.$$

In particular, we have $<s_i^{(n)} \cdot s_i^{(n+1)}> = \mu_i^2 + \varrho_i\sigma_i^2$ and $<\alpha^{(n)} \cdot \alpha^{(n+1)}> = \mu_\alpha^2 + \varrho_\alpha\sigma_\alpha^2$. We thus have:

$$\varrho_d = \frac{<dS_d^{(n)} \cdot dS_d^{(n+1)}>}{\sigma_d^2} = \frac{<s_i^{(n)} \cdot s_i^{(n+1)}><\alpha^{(n)} \cdot \alpha^{(n+1)}> - \mu_d^2}{\sigma_d^2} = \frac{\varrho_i\eta_i^2+\varrho_\alpha\eta_\alpha^2+\varrho_i\varrho_\alpha\eta_i^2\eta_\alpha^2}{\eta_i^2+\eta_\alpha^2+\eta_i^2\eta_\alpha^2}.$$

When α is deterministic (*i.e.* η_α=0), we have $\rho_d = \rho_i$: cell size homeostasis is determined by the initiation size homeostasis. On the other hand, when s_i is deterministic (*i.e.* η_i=0), we have $\rho_d = \rho_\alpha$. In general, the level sets of ρ_d are determined by a quadratic equation of the variables ρ_i and ρ_α. Therefore each level set is a conic section.

In the absence of mother/daughter correlations, namely $\rho_i = \rho_\alpha = 0$, then $\rho_d = 0$, which is a sizer regime. If we only have $\rho_i = 0$, then:

$$\varrho_d = \frac{\varrho_\alpha \eta_\alpha^2}{\eta_i^2+\eta_\alpha^2+\eta_i^2\eta_\alpha^2} .$$

For the IA model, we have $\rho_i = 1/2$. Furthermore, there are no mother/daughter correlations among the other physiological variables, therefore $\rho_\alpha = 0$. We thus obtain:

$$\varrho_d^{IA} = \frac{1}{2}\frac{\eta_i^2}{\eta_i^2+\eta_\alpha^2+\eta_i^2\eta_\alpha^2} < \frac{1}{2} .$$

Therefore, the IA model can only reproduce the adder correlation in the deterministic limit where $\alpha = \exp(\lambda\tau_{cyc})$ is a deterministic variable, namely $\eta_\alpha=0$.

Appendix D: Calculation of $\rho(s_i, S_d)$ and $\rho(S_d^{(n)}, S_d^{(n+1)})$ for the CCCP model

For simplicity, and following the conventions used by the authors of this model (14), we rewrite Eq. (8-11) as:

$$\ln(s_i^{(n+1)}) = q_i^{(n+1)} = \varrho_i q_i^{(n)} + A^{(n)} ,$$

$$\ln(S_R^{(n)}) = q_R^{(n)} = q_i^{(n)} + \lambda C^{(n)} ,$$

$$\ln(S_H^{(n)}) = q_H^{(n)} = \varrho_H q_H^{(n-1)} + B^{(n)} ,$$

$$\ln(S_d^{(n)}) = q_d^{(n)} = \max(q_R^{(n)}, q_H^{(n)}) ,$$

where we have introduced ρ_i and ρ_H to be more general. Assuming that $A^{(n)}$, $B^{(n)}$ and $C^{(n)}$ are Gaussian variables with means μ_A, μ_B and μ_C, and variances σ_A^2, σ_B^2 and σ_C^2, then $q_i^{(n)}$, $q_R^{(n)}$ and $q_H^{(n)}$ are also Gaussian variables. We first compute their means and variances. We have:

- $q_i \equiv N(\mu_i, \sigma_i)$ with $\mu_i = \frac{\mu_A}{1-\varrho_i}$ and $\sigma_i^2 = \frac{\sigma_A^2}{1-\varrho_i^2}$;
- $q_R \equiv N(\mu_R, \sigma_R)$ with $\mu_R = \mu_i + \lambda\mu_c$ and $\sigma_R^2 = \sigma_i^2 + \lambda^2\sigma_c^2$;
- $q_H \equiv N(\mu_H, \sigma_H)$ with $\mu_H = \frac{\mu_B}{1-\varrho_H}$ and $\sigma_H^2 = \frac{\sigma_B^2}{1-\varrho_H^2}$.

Note that q_H is independent from q_i and q_R. We now compute the autocovariances for these 3 variables. We obtain:

- $< dq_i^{(n+1)} dq_i^{(n)} > = \varrho_i \sigma_i^2$;
- $< dq_R^{(n+1)} dq_R^{(n)} > = \varrho_i \sigma_i^2$ since $C^{(n)}$ is an independently distributed random variable;
- $< dq_H^{(n+1)} dq_H^{(n)} > = \varrho_H \sigma_H^2$,

where as before $dX^{(n)} = X^{(n)} - < X >$. We furthermore compute for subsequent use the following cross-correlation: $< dq_R^{(n)} dq_i^{(n)} > = \sigma_i^2$ since $C^{(n)}$ is an independently distributed random variable. These preliminary results will be useful to compute ρ_d, ρ_i and ρ_{id}.

We now follow the approach of the authors, and replace the expression of q_d in Eq. (11) by the effective equation:

$$q_d^{(n)} = q_H^{(n)}u^{(n)} + q_R^{(n)}(1-u^{(n)}) ,$$

where $\{u^{(n)}\}$ are independent Bernoulli variables such that: $Pr(u^{(n)} = 1) = f$. We note that $Pr(u^{(n)} = 0) = 1-f$ and $Pr((u^{(n)})^2 = 1) = f$. The previous equation is an effective approach in which f represents the fraction of the cases in which the division process is limiting, namely $q_R^{(n)} < q_H^{(n)}$. With this effective expression, one can compute the mean μ_d and the variance σ_d^2 of q_d. We obtain:

- $\mu_d = \mu_H f + \mu_R(1-f)$;
- $\sigma_d^2 =< (q_H^{(n)})^2 >< (u^{(n)})^2 > + < (q_R^{(n)})^2 >< \left(1-u^{(n)}\right)^2 > + 2 < q_H^{(n)} >< q_R^{(n)} >< u^{(n)}\left(1-u^{(n)}\right) > - \mu_d^2$,
  $= \sigma_H^2 f + \sigma_R^2(1-f) + f(1-f)(\mu_H - \mu_R)^2$.

We now turn our attention to the computation of ρ_{id}. For that purpose, we compute the average:

$$< q_i q_d >=< q_i q_H >< u >+< q_i q_R >< 1-u >= \mu_i \mu_H f + (< dq_i dq_R >+ \mu_i \mu_R)(1-f) = \mu_i \mu_d + \sigma_i^2 (1-f) .$$

We thus obtain:

$< dq_i dq_d >=< q_i q_d >- \mu_i \mu_d = (1-f)\sigma_i^2$ and therefore:

$$\varrho_{id} = \frac{<dq_i dq_d>}{\sigma_i \sigma_d} = (1-f)\frac{\sigma_i}{\sqrt{\sigma_H^2 f + \sigma_R^2(1-f)+f(1-f)(\mu_H-\mu_R)^2}} .$$

We now turn our attention to the computation of ρ_d. We start by computing the average:

$$\begin{aligned} < q_d^{(n)} q_d^{(n+1)} >&=< q_H^{(n)} q_H^{(n+1)} >< u^{(n)} >< u^{(n+1)} >+< q_R^{(n)} q_R^{(n+1)} >< 1-u^{(n)} >< 1-u^{(n+1)} > \\ &+< q_H^{(n)} >< q_R^{(n+1)} >< u^{(n)} >< 1-u^{(n+1)} >+< q_H^{(n+1)} >< q_R^{(n)} >< u^{(n+1)} >< 1-u^{(n)} > , \\ &= \mu_H^2 f^2 + \mu_R^2 (1-f)^2 + 2\mu_H \mu_R f(1-f) + \varrho_H \sigma_H^2 f^2 + \varrho_i \sigma_i^2 (1-f)^2 . \end{aligned}$$

We therefore obtain:

$$\begin{aligned} < dq_d^{(n)} dq_d^{(n+1)} >&=< q_d^{(n)} q_d^{(n+1)} >- \mu_d^2 \\ &= \varrho_H \sigma_H^2 f^2 + \varrho_i \sigma_i^2 (1-f)^2 . \end{aligned}$$

Finally, we obtain:

$$\varrho_d = \frac{<dq_d^{(n)} dq_d^{(n+1)}>}{\sigma_d^2} = \frac{\varrho_H \sigma_H^2 f^2 + \varrho_i \sigma_i^2 (1-f)^2}{\sigma_H^2 f + \sigma_R^2(1-f)+f(1-f)(\mu_H-\mu_R)^2} .$$

When the division process is limiting, namely *f=1*, then we have $\rho_d=\rho_H$. Conversely when the initiation process is limiting, we have $\rho_d=\rho_i$. More generally, the level set curves for ρ_d are lines in the (ρ_H,ρ_i) plane.
